# Clinical outcomes of interactive, intensive and individual (3i) play therapy for children with ASD: a two-year follow-up study

**DOI:** 10.1186/s12887-018-1126-7

**Published:** 2018-05-12

**Authors:** Elodie Tilmont Pittala, Yann Saint-Georges-Chaumet, Claire Favrot, Antoine Tanet, David Cohen, Catherine Saint-Georges

**Affiliations:** 1 Cabinet privéé, 16 Avenue de la gares, 91570 Bievre, France; 2 Bioredac, 97 grande rue, 78240 Chambourcy, France; 3 Regional Psychiatric Center for Child and Adolescent with Deafness, 1st intersecteur, 64 rue de la glacière, 75013 Paris, France; 4 Department of Child and Adolescent Psychiatry, Hôpital de la Pitie-Salpêtriere, University Pierre and Marie Curie, 75013 Paris, France; 5 Institut des Systèmes Intelligents et de Robotiques, CNRS UMR 7222, Université Pierre et Marie Curie, Paris, France

**Keywords:** Autism spectrum disorders, Developmental intervention, Play-therapy, 3i method

## Abstract

**Background:**

The outcomes of psycho-educational interventions for Autism Spectrum Disorders (ASDs) comorbid with severe to moderate intellectual disability (ID) are insufficiently documented. In this prospective study, we examined a developmental individual, interactive and intensive approach, called the ‘3i method’, which is based on play therapy.

**Methods:**

Twenty DSM-IV-TR ASD subjects (mean chronological age 63.8 ± 37.8 months; mean developmental age 19.5 ± 6.6 months) were included and followed the 3i method for 24 months. Developmental and behavioural skills were assessed at baseline and after 24 months using the VABS, PEP-R and Nadel Imitation scale. Autism severity was evaluated using the Child Autism Rating Scale (CARS) and the Autism Diagnostic Interview (ADI-R).

**Results:**

After 2 years of the 3i method, our 3 primary outcome variables significantly increased (VABS developmental age of socialization increased by 83%, age of communication by 34%, and Nadel Imitation score by 53%). Almost all VABS and PEP-R domains significantly improved. Additionally, increases in the VABS socialization score were positively correlated with the total number of treatment hours and CARS score; all ADI-R areas significantly decreased; and diagnoses had changed in 47.5% of the subjects (37% for PDD-NOS and even 10.5% for ID without PDD).

**Conclusion:**

Children who followed the 3i method for 2 years had significantly improved behavioural and developmental skills and showed a clear decrease in autism severity. These results suggest that the 3i method may be useful for autistic children by improving their daily interactions with their social environment.

**Trial registration:**

was retrospectively registered on May 20th, 2014 by the French Agency for drug and health (ANSM) under number ID-RCB 2014-A00542–45, reference: B148558–31.

**Electronic supplementary material:**

The online version of this article (10.1186/s12887-018-1126-7) contains supplementary material, which is available to authorized users.

## Background

Autism spectrum disorder (ASD) is a life-long neurodevelopmental disorder characterized by early impairments in socio-communicative skills that are associated with a set of restricted interests and/or repetitive stereotypical behaviour [1]. Recently, the fifth edition of the Diagnostic and Statistical Manual of Mental Disorders (DSM V) added sensory impairments to the diagnostic criteria of the behavioural manifestations of ASD [2–4]. Many research efforts have been made to identify precursors of ASD, leading to a significant decrease in the age of diagnosis [5], which enables earlier treatment. The existing treatments are mainly symptomatic.

***Behavioural methods,*** which aim to develop certain expected behaviours through child reinforcement, have been proposed to treat ASD with significant scientific support [6, 7]. Applied Behaviour Analysis (ABA) is a one-to-one intensive approach based on behavioural strategies and targets specific symptoms using reinforcement of adaptive, expected skills [8, 9]. After criticisms regarding the lack of spontaneity in this early approach, Pivotal Response Training was subsequently developed and uses children’s own choice of games and activities to reinforce the correct answers expected by the professional; this type of training also favours providing complete or incomplete forms of attempts to respond, alternating between acquisition and maintenance, and using intrinsic reinforcers [10]. Additionally, the Treatment and Education of Autistic and related Communication handicapped CHildren (TEACCH) program emphasizes a close working relationship between parents and practitioners, adapts the intervention to the characteristics of the child and uses structured teaching educational techniques [11].

Alternatively, ***developmental approaches*** start from the specific interests and resources of the child and aim to restore the global developmental process to increase communication skills and learning ability. One example is the Son-Rise Program®, developed in the early eighties, which was essentially based on “joining” the child, playing with him and following his cues. This program has been followed by tens of thousands of families in the U.S.A., and a recent study based on 5 days of intensive treatment of 6 children showed encouraging results [12]. Other developmental interventions based on play therapy have conceptualized the relationship between playing and child development, such as Floor Time [13, 14] or in France, “Exchange and Development Therapy” (EDT), which was developed in the 1990s by Lelord [15]. Floor Time consists of sequences of guided play (15–20 min) that are repeated several times by parents throughout the day and are supervised by an expert. Floor Time is the core of the Developmental, Individual Differences, and Relationship-based (DIR) method. DIR focuses on individual developmental differences in a child’s emotional functioning, information processing, motor planning and types of interactions. The DIR method recommends following the child’s lead and supporting his/her initiative; focusing on joint attention; closing circles of communication; creating semi-structured problem solving approaches; contrasting repetitiveness with playful obstruction; supporting visual attention; and working on imitation [16, 17].

**Some methods mix elements** of developmental play therapy with a structured and behavioural teaching approach**.** The Braintraining method [18] associates guided play time with teaching material depending on the specific developmental stage of the child’s playing ability. Multi-sensory activities help increase the child’s level of multimodal integration, overcoming difficulties that would restrict the development of more sophisticated cognitive skills such as symbolic play, language and social understanding. The Early Start Denver Model (ESDM) is an early and intensive intervention approach for young children that combines developmental and behavioural approaches. This method was evaluated in a randomized control trial with 48 toddlers [19], and the study showed that ESDM effectively improved cognitive and adaptive behaviour and reduced the severity of ASD diagnoses. A complementary electro-physiologic study suggested that a certain normalization of brain function was associated with clinical improvement [20]. In recent years, the ESDM has aroused great interest; for example, a study on at-risk infants suggested increased developmental abilities and a decrease in autism severity after 3 years of ESDM treatment [21]. Some behavioural therapists have also tried stimulation with small children using their favourite games and activities and showed a sustained improvement in social interactions [22]. Thus, the concept of triggering developmental processes in children with ASD has advanced in the scientific community.

In France, health facilities often provide institutional approaches to develop relational skills through various activities and therapeutic mediations, in groups or individually [23]. These approaches have increasingly been associated with structured tools from an integration perspective; for example, the ESDM has recently been integrated into the French health care service [24]. A previous review highlighted the diversity of practices in France but also the lack of published data on the effectiveness of these institutional strategies [25]. A French multicenter study is currently underway to evaluate the effectiveness of an integrated, individual, intensive structured teaching program provided to children with ASD and moderate intellectual disability (ID) in institutional care [26].

Beyond the diversity of individual approaches, an analysis of the literature has provided some important guidelines for improving the treatment of ASD children [27], including the following: i) Diagnose and start interventions early; ii) provide at least three or 4 hours of treatment each day; iii) center interventions on family involvement, placing parents in a position that allows them to actively participate in the education of their child; iv) provide regular updates on progress and subsequent goals; v) choose between behavioural or developmental treatment depending on the child’s response; vi) encourage spontaneous communication; vii) include interactions with peers as soon as possible; viii) help extend the acquired skills to various environments and natural contexts; and ix) support positive behaviours rather than tackling challenging behaviours [25, 27]. Notably, previous studies suggested that involving the family in the treatment of the child promoted the therapeutic effect. For example, in a French study on a 20-month home-intensive method involving the family, 25 children progressed from 6 to 25 points in their developmental quotient [28]. In addition, a randomized controlled Australian study showed that parental involvement through home-specific work promoted the efficiency of treatment [29, 30]. Similar results, showing beneficial effects of parental contribution in early intervention, have been published and highlight the importance of parental involvement in treating ASD [30–32], even for long-term outcomes [33].

In this study, we examined a developmental, Individual, Interactive and Intensive approach to treat ASD comorbid with ID called the ‘3i method’, which was based on the playing ability of the child and included the family. As this play therapy was meant to stimulate developmental processes, the child’s progress was analysed by a psychologist through regular team meetings, considering the normal developmental pattern of a child of his/her developmental age. Here, we present a prospective exploratory study that aims to assess the outcome of 20 ASD subjects who followed the 3i method for 24 months. Using appropriate scales, we estimated the course of developmental and behavioural skills and autism severity after 2 years.

## Methods

### Participants - ethics

This study’s protocol was approved by the institutional ethical committee and was retrospectively registered on May 20th, 2014 by the French Agency for drug and health (ANSM) under number ID-RCB 2014-A00542–45, refence: B148558–31 (Additional file 1: Protocol). All parents of the subjects spontaneously contacted the *Association Autisme Espoir vers l’Ecole* (AEVE) to help them provide 3i treatment for their children. In total, 37% of the parents discovered AEVE through its website, 38% through relatives, 16% through meetings or other media and 9% through health caregivers. The parents voluntarily provided informed consent to include their child in the study after being informed of the study design and goals.

The inclusion criteria were as follows: being diagnosed with ASD based on the Autism Diagnostic Interview, Revised (ADI-R) and the Childhood Autism Rating Scale (CARS); initiating the 3i method at some point between January 2013 and December 2013; residing in an environment that allowed for the correct application of the 3i method (possibility of a dedicated room, ability to recruit and keep volunteers, as determined by the AEVE’s headmaster); not receiving any other ASD intervention treatment method (e.g.*,* ABA or TEACCH); and having French as their maternal language. The exclusion criteria were as follows: the presence of associated diseases such as epilepsy or Rett syndrome; Asperger’s syndrome; and the inability to ensure 3i treatment for the entire duration of the study. Patients aged > 15 years old at the beginning of the study were not included. Asperger patients, with their outstanding intellectual abilities, were not considered for this study as a standard 3i program would need to be customised to their abilities, and the volunteers would need a special training.

During the one-year selection period (January to December 2013), 31 children began the 3i program, but only 20 were included in this study. Four of the eleven remaining children were outside the age range (too old), three presented Asperger’s syndrome, two suffered from epilepsy, and two could not receive intensive 3i treatment. All the included subjects were not attending school and followed the 3i method between 30 and 35 h per week. Specifically, 17 children followed the 3i method at home and 3 at the AEVE Courbevoie day centre. The patients’ age at the beginning of the study ranged between 2.8 and 14.5 years.

### 3i method

The 3i method is promoted by AEVE, a non-profit association founded in 2006. The 3i method is derived from the SonRise® Program but has important differences. Similar to SonRise®, the intervention occurs in a specific room devised for one-on-one interaction and designed to reduce unwanted sensorial stimuli. The basic intervention principles are also the same and are directed towards “joining” the child’s world without any expectations through playful reactions to the child’s cues [34]. However, the 3i method also i) focuses on the sensory specificities of the child, ii) provides participants with a developmental roadmap that improves understanding of the present abilities and difficulties on their developmental path, and iii) distinguishes three developmental age stages in their corresponding agenda (0 to 18 months, 18 to 36 months and older than 36 months [35]).

The 3i method presents important similarities with Exchange and Development Therapy (EDT), which is practised in France and is based on approximately two 45-min sessions each week [15]. The key aspects of this therapy are as follows: i) a quiet environment to limit excessive neural reactivity in the child to sensorial stimuli; ii) openness of the therapist, lack of expectations, and careful observation; and iii) facilitation of reciprocity by, for example, free imitation [36].

However, the 3i method is recommended to be intensive: a minimum of 30 h of play sessions per week in a specific playroom are proposed. Each game session lasts 1 and one-half hours. The recommended size of the playroom is approximately 10 sq.m., which offers a space with boundaries visible by the child. The lighting must be subdued, and the sounds should be muffled with appropriate ground lining. The standard equipment in the room includes shelves out of the child’s reach where objects will be visibly stored, a mirror, a swing and a few other items used to gain a physical perception of oneself; these items can be visual such as clay, tactile such as colour cubes, and auditory such as a triangle or xylophone, in addition to picture books, puppets, etc. The sessions are conducted by volunteers. These participants are mostly non-professionals in the field of ASD. Volunteers are first screened by the parents and then participate in a meeting with the head of AEVE, who provides them with information regarding autism and the 3i method. They are screened based on their ability to ensure good care for children before beginning to interact with the child in the playroom. They subsequently participate each month in a group session with the other participants, together with the parents, under the supervision of a 3i-trained psychologist (who is paid by the parents). For each child, this 3i-trained psychologist manages the team of volunteers and ensures the consistency of their actions and their compliance with the 3i method. The child’s progress is recorded and analysed by the psychologist, who provides some advice according to the participants’ questions and the child’s evolution.

The 3i intervention is organized into three phases that correspond to the developmental age brackets of 0 to 18 months (Phase 1), 18 to 36 months (Phase 2), and over 36 months (phase 3). During phase 1, the intervention is centred on simple sensorial games and exchanges, without recourse to many objects. The aim is to help children discover themselves, their body and the distinct existence of another person. During phase 2, the child’s attention is brought to the external world outside the playroom, a meaningful language emerges, and the child gains access to symbolic play. The child’s desire for actual conscious learning appears in phase 3 and leads to progressive integration into a classroom as a classmate, without being seen as a handicapped child. The 3i method has been used in different countries, particularly in Poland, where the 3i method has been extensively described [35, 37]. The method was also carefully described in a recent retrospective study investigating the effect of 2 years of the 3i approach on the socio-communicative skills of 120 ASD children (Favrot C, Saint-Georges-Chaumet Y, Saint-Georges C: Evaluation of the efficiency of the 3i method from a retrospective archive study on 120 children diagnosed with Autism Spectrum Disorders, submitted). In addition, a retrospective single case study with 10 years of follow-up shows the long-term positive effects of the method (Astrup O: An individual study of an autistic child over a nine years period using the 3i method, in preparation). Despite these different sources of information, the method has not yet been manualized.

### Clinical variables

Three variables were chosen as the primary measures: developmental age in the Vineland Adaptive Behaviour Scale (VABS), communication and socialization scores and the Nadel imitation scale (NIS) score [38]. The secondary outcomes were the developmental ages of imitation and verbal communication according to the Psycho-Educational Profile – Revised (PEP-R) scale and the severity of autism according to the CARS. The ADI-R was also used to assess diagnosis status and eventual changes.

The VABS is a measure of adaptive behaviour [39] that was designed to assess handicapped and non-handicapped persons (birth to 18 years and low functioning adults) in their personal and social functioning. Split-half and test-retest reliability coefficients for the composite scores are good, ranging from median values of 0.83 for the motor skills domain to 0.94 for the composite score. Interrater coefficients are lower for the same measures: 0.62 to 0.78 [40]. For validity, correlations with concurrent measures (the original Vineland, the ABIC, the K-ABC, and the PPVT-R) were shown to be low to moderate, with generally higher coefficients obtained when the comparisons were made on subjects with handicapping conditions. We used it here to assess the impact of the 3i method on the development of socialization, communication, and autonomy in everyday life. Since we do not have tables for converting raw scores into standard scores from sampling in the French population, the results of the evaluations are expressed in months of developmental age. The NIS [38] was added as a primary variable because we aimed to include children with severe intellectual disability as well (developmental age < 18 months), for whom the NIS is better suited, because imitation is a very early skill in development. The NIS provides preverbal communication scores, with a focus on imitation. It contains three items: spontaneous imitation, recognition of emulation and imitation on request. An analysis of the scores obtained in the three evaluation times (T0, T1, and T2) offers finer monitoring of the impact of the 3i method on the development of non-verbal communication, particularly in very young or nonverbal subjects.

The PEP-R test is used to calculate the patient’s developmental age at different evaluation times in the following developmental areas: imitation, perception, fine motor skills, global motor skills, oculo-manual coordination, cognitive performance, and verbal cognition according to behavioural observations [41]. A developmental age can be calculated for each of these areas. PEP-R psychometrics were studied in a sample of 116 children with the dual disability of an intellectual disorder and autism. PEP-R demonstrated a good internal consistency (Cronbach’s α ranging from 0.91 to 0.93) and domain total correlation (ranging from 0.75 to 0.90). The interrater reliability (intraclass correlation coefficient, ICC = 0.96) and test-retest reliability (ICC = 0.87) were good, and there was moderate-to-high concurrent validity with Gesell’s Developmental Schedule (r ranging from 0.61 to 0.82; all *P* = 0.001) [42]. In this study, we specifically focused on the imitation and verbal cognition developmental ages because they were deemed more significant prerequisites of successful school (re)entry.

The ADI-R is a diagnostic assessment tool based on the description and history of the patient that analyses his/her development in three areas: quality of social interactions, communication and language, and restricted interests and stereotyped behaviours [43]. The ADI-R has good reliability and validity and is currently considered the gold standard for diagnosing children with autism for research purposes. We chose to use the ADI-R rather than the ADOS 2 because it allows a more global evaluation of children’s abilities in various ecological contexts and not only in the context of a formal evaluation with an unknown observer. Parents are often very informative because they are close observers of their child’s behaviours. Although parental reporting could lack objectivity, other tools such as the CARS and PEP-R were scored through an external observation; thus, ADI-R appeared complementary to these spot observations. The ADI-R was used 3 times during this study as a secondary criterion to assess the efficacy of the method.

The CARS was developed to identify and classify children with *autism* and to determine symptom severity through quantifiable ratings based on direct observation. CARS interrater reliability is good from 0.55 to 0.93, and for validity, the CARS yielded results consistent with the judgements of clinical experts [44]. The following 15 items assessed are those typically disrupted in autism: social interaction, imitation, emotional response, body use, use of objects, adaptation to change, visual responses, auditory responses, taste-smell-touch (and answer mode exploration), fear and anxiety, verbal communication, nonverbal communication, activity level, intellectual level and homogeneity of intellectual functioning; finally, a global perception of the subject is generated. CARS was used in this study to characterize the intensity of autistic disorder and its evolution during treatment.

### Study design

Evaluations were conducted at the usual treatment location. Developmental and behavioural skills assessments with the PEP-R, VABS and NIS were performed by the first author and another psychologist through an observational session and a parental interview within a month after the first day of treatment (T0), 1 year after beginning the treatment (T1) and finally 2 years after beginning the treatment (T2). In addition, the severity of the autistic syndrome was evaluated through the ADI-R (parental interview administered by 3 external psychologists not working with the child) and CARS (some by external psychologists, others by the psychologist who supervised the method) at the beginning (T0) and end of the study (T2) (Table 1).

**Table 1:** Calendar of outcome assessments

	T0 (+ 1 month)	T1 (+ 12 months)	T2 (+ 24 months)
ADI-R	x	x	x
CARS	x	x	x
VABS	x	x	x
PEP-R	x	x	x
IMITATION (Nadel)	x	x	x

During the 2-year follow-up, the total duration of participation (patient and parent) in the specific assessments was approximately 22 h. The evaluations occurred in the child’s usual playroom and in a place suitable for parental interviews. To optimize the evaluation sessions, the playroom was designed to exclude anything that could distract the patient during the assessment (slide, swing, mirror, etc.). The playroom was equipped with only two chairs and a table, two cameras on tripods and the evaluation materials.

### Statistics

Three subjects left the study before undergoing the T2 tests for the VABS, PEP-R, NIS, CARS and ADI-R. The T2 missing values were inferred by replacing their T2 with T1 values in accordance with the “Last Observation Carried Forward” method [45]. The low data homogeneity and small number of subjects implied the use of non-parametric tests to assess differences between the three evaluation times. A multiple comparison of paired measures was performed with Friedman’s test. A post hoc analysis (to determine which group of measures differed from the others) was performed if the Friedman test was positive with the Bonferroni correction. *P*-values were considered significant at *p* < 0.05. Statistical analyses were performed using R software version 3.2.1.

## Results

### Subjects

Twenty subjects were enrolled in the present study between January 2013 and March 2014. All participants were diagnosed with ASD according to the DSM-IV-TR criteria [46]. More specifically, according to the ADI-R scores, all subjects met the criteria for autistic disorder [43]. Boys represented 90% (18 out of 20) of the study sample (Table 2). The chronological age at the starting point varied between 33 and 173 months. However, the developmental ages calculated with the PEP-R test were more homogeneous, with a median developmental age of 19.5 ± 6.6 months (minimum 13, maximum 38). Additionally, 65% (13 out of 20) of the subjects were initially categorized as nonverbal with regard to their mode of communication. Overall, the subjects spent 2832 ± 550 h following the 3i method during the 2 years of their study period. This means that every patient had an average of 4 h of 3i sessions per day. All twenty subjects completed the baseline and first year’s evaluations (T0 and T1). Three subjects dropped out after 1 year. One subject was placed by his parents into a medico-educative institution due to substantial progression. One patient dropped out because the parents rejected pursuit of the 3i method at home. The last patient was lost to follow-up. The results of developmental and behavioural skills and ASD severity are summarized in Tables 3 and 4, respectively. Individual results with the chronological age of the patients are presented in Additional file 2: Table S1.

**Table 2:** Sample description at baseline

Characteristics of subjects at inclusion	
Number	20
Sex	Boys: 18; Girls: 2
Chronologic age (months ± SD)	63.8 ± 37.8
Developmental age (month ± SD, from global PEP-R score)	19.5 ± 6.6
Developmental quotient (mean ± SD from global PEP-R developmental age)	35.3 ± 13.1
Type of communication (%)	Nonverbal: 13 (65%)
	Gestures: 1 (5%)
	Babbling: 2 (10%)
	Verbal: 4 (20%)
Total duration of treatment (hours ± SD)	2832 ± 550

**Table 3:** Variation in developmental and behavioral skills throughout the study

Evaluation	T0	T1	T2	P-value Friedman
VABS Communication (mean ± SD)	15.8 ± 9.0	19.9 ± 10.0***	21.7 ± 11.2***	1.2 10^−6^
VABS Socialization (mean ± SD)	12.8 ± 6.5	19.2 ± 7.6***	24.3 ± 13.4***	2.7 10^− 6^
Imitation Score Nadel (mean ± SD)	9.6 ± 5.7	13.4 ± 7.1**	14.3 ± 6.3**	6.5 10^−5^
PEP-R Imitation (mean ± SD)	11.2 ± 8.1	16.0 ± 11.8*	18 ± 15.0**	4.5 10^− 5^
PEP-R Verbal cognition (mean ± SD)	17.6 ± 9.4	22 ± 13.0	20.9 ± 16.0	0,207
Global PEP-R (mean ± SD)	25.7 ± 10.5	30.9 ± 13.5**	33.5 ± 17.0***	5.83 10^− 6^
PEP-R Perception (mean ± SD)	28.5 ± 12.6	38 ± 17.7**	40.5 ± 17.1**	4.48 10^− 4^
PEP-R Fine motor skills (mean ± SD)	30.6 ± 14.7	35.2 ± 15.0*	39.7 ± 17.2*	0.0147
PEP-R Global motor skills (mean ± SD)	32.3 ± 12.9	41.0 ± 14.4**	34.0 ± 20.3	8.59 10^− 3^
PEP-R Oculo-manual development (mean ± SD)	28.8 ± 14.5	35.5 ± 16.5*	38.8 ± 18.2**	1.12 10^− 3^
PEP-R Cognitive performance (mean ± SD)	21.9 ± 14.8	25.5 ± 15.2	26.9 ± 19.0	0.0293
VABS Autonomy (mean ± SD)	23.1 ± 9.7	27.2 ± 10.3***	32.1 ± 12.4***	9.87 10^− 7^
VABS Motricity (mean ± SD)	31.6 ± 11.5	37.5 ± 12.4**	38.5 ± 14.1**	2.31 10^− 4^

All scores are developmental age in months except for the imitation score based on the Nadel scale. The * indicates the level of the *p*-values of the post hoc tests between T0 and T1 and T0 and T2 after the Friedman test. Bonferroni adjustments were made due to the multiple analyses *** *p* < 0.001; ** *p* < 0.01; * *p* < 0.05

**Table 4:** Assessment of autism severity during the study

Evaluation	T0	T1	T2	*P*-value
CARS	44.5 ± 5.0	37.1 ± 5.6**	33.7 ± 5.1***	1.76 10^−7^
ADI-R Interaction	23.1 ± 3.2	14.6 ± 3.8***	12.7 ± 5.0***	1.21 10^− 7^
ADI-R Communication	13.0 ± 3.7	10.6 ± 3.1*	8.3 ± 2.8**	4.77 10^− 5^
ADI-R Stereotypy	6.4 ± 2.6	5.1 ± 2.5	4.0 ± 2.1*	0.00235

The * indicates the level of the p-values of the post hoc tests between T0 and T1 and T0 and T2 after the Friedman test. Bonferroni adjustments were made due to the multiple analyses *** *p* < 0.001; ** *p* < 0.01; * *p* < 0.05

### Developmental and behavioural skills

**Fig. 1:**
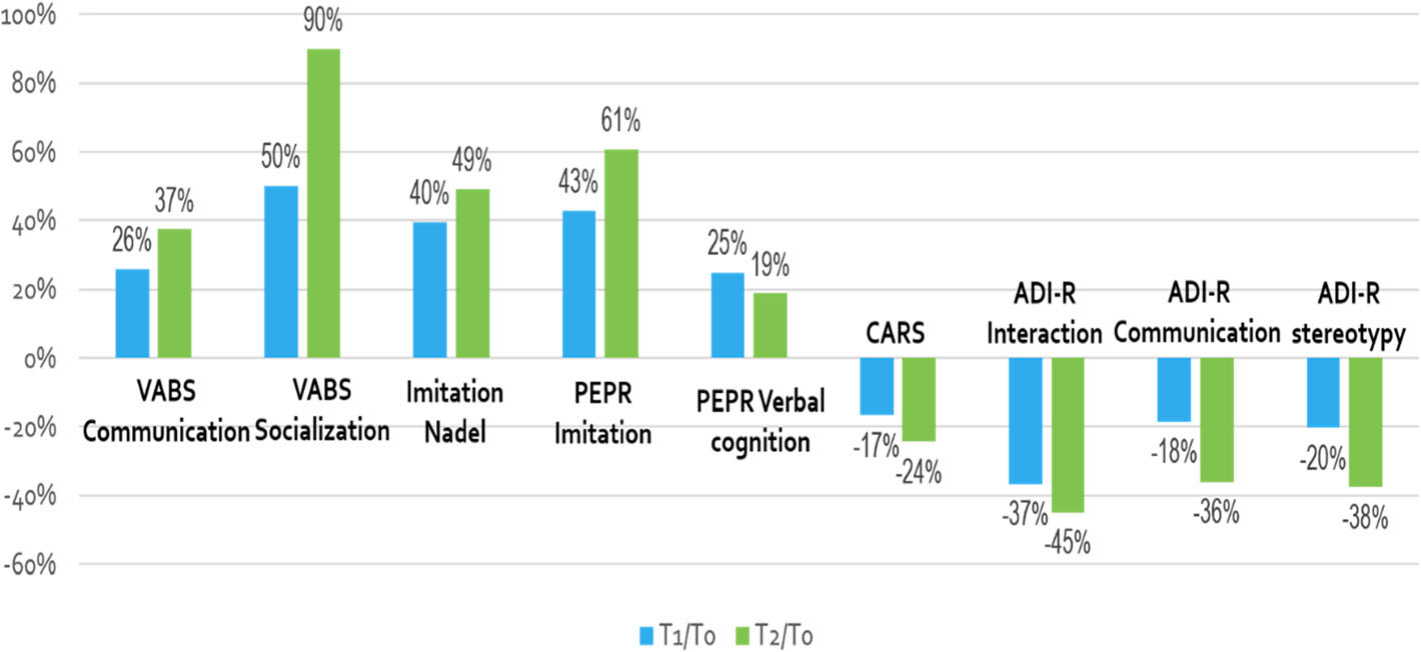
Graph of the progression in behavioral and developmental skills and autism severity among subjects. The changes in median scores of the VABS developmental age. PEP-R developmental age. Nadel imitation scale and the CARS and ADI-R evaluations are reported according to the T0 values and expressed as a percentage

All 3 principal outcomes increased significantly during the 2 years of the 3i method (Table 3, Fig. 1). The developmental age of communication according to the VABS increased significantly from 15.8 ± 9.0 to 21.7 ± 11.2 months, representing a 34% increase. Additionally, the developmental age of socialization according to the VABS increased significantly from 12.8 ± 6.5 to 24.3 ± 13.4 months, representing an 83% increase after 2 years of the 3i method. The NIS score increased significantly by 53%, which is consistent with the 67% increase after 2 years of the 3i method in the PEP-R imitation developmental age (6.8 months). The PEP-R verbal cognition score increased by only 23% after 24 months, which was not significant.

Additionally, of the 8 other items covered by the VABS and PEP-R evaluations, all increased significantly during the 24 months of the study, except for PEP-R global motor skills (Table 3). The overall results suggest that 24 months of the 3i method was associated with a global increase in developmental and behavioural skills among our 20 subjects.

### ASD severity

In addition to developmental and behavioural skills, we assessed variation in ASD severity using the CARS and ADI-R with 19 patients (unfortunately CARS and ADI-R data at T1 and T2 are absent for one patient) (Table 4, Fig. 1 and Additional file 2: Table S1). Based on these results, the mean CARS score dropped significantly from 44.4 ± 5 to 33.7 ± 5.1 at the end of the 2 years. A CARS score > 37 indicates severe autistic disorder; a score between 30 and 37 indicates slight to moderate autistic disorder; and scores under 30 indicate patients who are outside the ASD range. Based on this scale, 94% of our patients were considered severely autistic at the beginning of the study and 6% were considered moderately autistic. At the end of the 2 years, only 21% (4/19) remained severely autistic, 53% (10/19) had progressed to a moderate autistic score, and 26% (5/19) could be considered to no longer have autism (Fig. 1). In addition, all ADI-R areas (interaction, communication, and stereotypy) significantly decreased during the 24 months of treatment (Table 4 and Figure 1). According to the ADI-R, 9/19 (47.3%) subjects had an improved DSM-IV diagnosis. Seven subjects’ diagnoses changed from autistic disorder (AD) to Pervasive Developmental Disorder-Not Otherwise Specified (PDD-NOS), and 2 subjects were no longer categorized as having PDD. All individuals still had moderate to severe intellectual disability at the end of the study.

### Prediction of outcomes

**Fig. 2:**
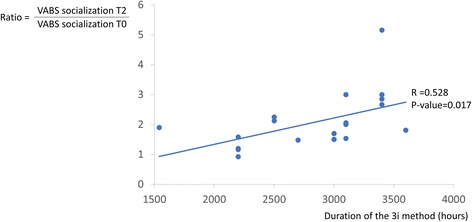
Plot of the duration of the 3I method (in hours) and the ratio of the VABS socialization score at T2 to the VABS socialization score at T0 (baseline). A positive significant positive correlation was found (*P*-value =0.017) between these two variables

To test whether any baseline variables were correlated with the developmental and behavioural skills’ evolution and the decrease in ASD severity observed, the correlations between each of the 3 principal outcome variables were tested with the following: the patient’s chronological age at the beginning of the study, the developmental age (based on the PEP-R) at the beginning of the study, the developmental ratio (developmental age/chronological age) at the beginning of the study, the total duration of treatment and the CARS and ADI-R scores at the beginning of the study. Only the total duration of the treatment was positively correlated with the VABS socialization score (Fig. 2). This latter result suggests that a greater number of hours of treatment predicted better improvement in the VABS socialization score.

No noticeable difference could be observed between the three patients treated with the 3i method in a centre compared to those treated at home (Additional file 2: Tables S1 and S2). In addition, the progress of younger patients (beginning the 3i method before 5 years of age) was not significantly different from that of older patients, except for the imitation score of Nadel (Additional file 2: Tables S1 and 3). However, because of the low number of patients, these results must be interpreted with caution. Indeed, the increases in VABS scores were higher for younger patients, particularly for the socialization VABS score, which displayed a significant differential progression.

## Discussion

Our sample consisted of a population of children who are rarely studied because they presented with severe ASD (mean CARS = 44.5, Table 1) with moderate to severe intellectual disability (mean developmental quotient based on PEP-*R* = 35.3 +/− 13.1) at the start of the study. In fact, numerous studies choose to exclude children with a developmental quotient (DQ) below 35 [19]. Our results showed that after 24 months of the 3i method, our sample of 20 patients globally progressed in different skills including socialization, communication, perception and imitation. In line with these developmental changes, the severity of ASD significantly decreased: after 2 years of treatment, 26% of the subjects moved below the CARS threshold proposed for ASD (Table 4). Accordingly, several ADI-R diagnoses changed: although all patients were initially diagnosed with autistic disorder (AD), after 2 years of the 3i method, 9 out of 19 (47%) had an improved diagnosis. These results appear to be encouraging, in particular because of the relatively old age of the subjects and the lower cognitive profile (mean DQ = 35) compared to well-designed randomized trials previously published [19].

Interestingly, the VABS socialization change ratio was positively correlated with the total duration of receiving the 3i method. This correlation may indicate one major effect of the 3i method. This variable was the only principal outcome that continued to significantly increase in T2 compared to T1. Indeed, the 3i method involves the use of multiple volunteers interacting several hours a day with the child. This intensive relationship may increase the socialization of the subjects in proportion to the hours spent in the playroom. This result may highlight one of the major interests of the 3i method for treating ASD children.

By contrast, no correlation between the subjects’ age and the overall decrease in ASD severity/diagnosis or their progress in developmental skills was found. We also compared the progress of children aged < 6 years (*N* = 16) with those of children aged > 7 years (*N* = 4). In accordance with the correlation test results, no statistically significant differences were observed between these two groups except for the imitation scale results (Additional file 2: Tables S1 and S3). This last result suggests that younger 3i patients may present a better improvement of imitation skills compared to older patients. However, the age effect should be retested with a larger cohort to increase the statistical force. Overall, chronological age does not appear to be a determinant of the success of the 3i method.

### Comparing the 3i (individual, interactive and intensive) method with other ASD children interventions

***Individualising*** **the intervention** through considering the developmental stage and specific needs of the child can occur via the one-on-one context and the focus on the child’s development through meetings with the psychologist of the 3i method. Studies on the Denver approach [19] support the idea that using a developmental perspective is more efficient. Our prospective study, similar to our previous retrospective study on 120 patients, is in line with this view.

#### Playing as a support for *interaction*

In terms of play therapies and the 3i method, Gardziel et al. previously commented on the positive aspects of the 3i method and described its beneficial effects on one ASD patient [35]. In another study, the progress of 3 ASD patients treated with the 3i method was assessed using the BECS, PEP-3 and CARS [47]. After 2 years of receiving the 3i method, the 3 children showed a positive evolution: they had less invasive autistic disorders and communicated and interacted more with others. Additionally, a retrospective single case study on a 2-year-old boy treated for 30 months with the 3i method reported a 9.5 point decrease in his CARS score, placing him below the threshold for autism; furthermore, his 3 ADI-R scores, which were all impaired at baseline, were shown to be out of the autistic diagnosis range when assessed after 10 years of follow-up (Astrup O: An individual study of an autistic child over a nine years period using the 3i method, in preparation). Finally, another retrospective study on 120 children treated for 2 years with the 3i method (Favrot C, Saint-Georges-Chaumet Y, Saint-Georges C: Evaluation of the efficiency of the 3i method from a retrospective archive study on 120 children diagnosed with Autism Spectrum Disorders, submitted) and assessed by a skill-building stage scale seems to be in line with our results: on average, the subjects acquired a higher stage of competence in four of the six skill areas (Imitation, Gaze Quality, Social and Emotional Regulation, Verbal Expression, Non-Verbal Expression and Verbal Comprehension), with a greater increase in the skills of imitation and non-verbal communication. The present study prospectively shows for the first time the benefit that ASD patients can receive from intensive play therapy with the 3i method. These results are in line with those of other studies on play therapy: Floor Time [14, 17], “Exchange and Development Therapy” [15], and SonRise [12]; ESDM also partially uses play therapy components. All these play therapy methods share a developmental vision in which the child can succeed in building relational abilities through repeated experiences in individual relationships with responsive, joyful and empathic adults, before learning to face other children. This primary dyadic context may be crucial for the development of the first stages of relational abilities. In the Floortime study, the 31 children who received 14 weekly hours of play with their parents progressed in skills within 1 year at the Functional Emotional Assessment Scale (FEAS) and at the Functional Emotional Developmental Questionnaire (FEDQ) and exhibited a mean decrease of 3 points in their CARS score. The SonRise study showed a significant effect of an intensive week (40 h) of treatment in 6 children (compared to 6 control children) on interaction and communication measures. All these studies support the idea of efficiency of stimulating through a play adapted to the developmental level of the child, and our results are in line with that view.

Play support, tailoring to child developmental level and structuration are aspects of the 3i method that are similar to “Exchange and Development Therapy” (EDT) [15]. Thirty-five children aged 2 to 7 years old treated with this method for a 9-month period improved their skills in imitation, joint attention and interaction (on the BECS scale) and exhibited a decrease in interaction and communication disorders (in the ECA-R). Although the mean age, scales and length of observation differ, these results are congruent with those of our study. However, although the EDT children had a few sessions per week and received other interventions simultaneously (day-care hospital, school inclusion, etc.), intensivity (at least 30 h a week) and the use of volunteers at home are specific in the 3i method. This study suggests that a developmental play-therapy method can be intensive without mobilizing many professionals and alone is able to allow the child to progress in key areas of development.

***Intensivity*** (several hours a day) is recommended by Narzisi [27] based on his literature review. Behavioural approaches and the Denver Method often stress this need for intensivity. Once autistic syndrome is installed and stable, repetition of therapeutic interactive experiences may be crucial to allow new functioning and new brain circuitry to develop and finally outgrow autistic functioning. This study, similar to the SonRise study [12], shows that play-therapy can be intensive and provides encouraging results.

##### Parental implication

Another interesting point that makes the 3i method different from SonRise or EDT is the importance of including parents in the therapy. In the 3i approach, parents must recruit volunteers, accommodate them at home and coordinate their interventions; they sometimes participate in planning the intervention themselves. They are involved in the care and thus are led to change their comprehension of their child and the quality of their daily interactions with them. As mentioned in the introduction, some studies have reported a positive impact of parental participation [29–32, 47]. Their inclusion may help extend the beneficial effects of treatment even outside the time allotted for therapy. Our study again underlines the benefit of involving parents. However, for the 3 patients who were treated in a centre instead of in their private homes, no significant differences were found in their outcomes compared to those of the 17 home-treated patients. These data suggest that the place of intervention may not affect the treatment outcome, but additional data are necessary with a larger sample to really assess this effect.

##### Voluntaries intervention

Interacting with ASD children represents the utmost difficulty. In the 3i method, the presence of volunteers, non-professionals and their large number is an asset, for several reasons. First, as a play-based method in which it is crucial to adapt to the level of the child and be creative, the large number of volunteers allows them to spend only 1.5 h twice a week and consequently not be exhausted by the bewildering behaviours of the child, allowing them to remain creative and at the child’s disposal. This short time spent with the autistic child could be a powerful factor to prevent the adult’s discouragement and the corresponding withdrawal effects that may occur in the child when he perceives the negative or depressed attitude of the adult. Second, it is an ecological framework because the team of volunteers consists of a few family relatives (parents and grandparents) and other adults chosen by the parents in their immediate environment. This allows the child to be part of his social and cultural life, favouring insertion and recognition in a community. Moreover, this team, which is quite diverse in age and sex, corresponds to the population that the child will encounter in ordinary life. The group of volunteers represents a first positive and warm social experience, allowing the child to socialize gradually in a friendly living environment.

##### Specificities and prospects for education

A progression to higher scores in our sample was observed in VABS socialization (90% mean increase), PEP-R imitation (+ 61%), Nadel imitation (+ 49%) and ADI interaction (− 45%). These results are consistent because socialization, imitation and interaction are domains that appear to be directly or indirectly linked. Interestingly, although these children had not been “socialized” at school, the greatest improvements were observed in socialization. Peer-to-peer immersion is thus not the only way to “socialize”, and an alternative method could be used to improve interactive and relational functioning in a privileged system with responsive adults before joining the school system. This suggests that first acquiring the ability to be in a relationship, before learning how to adapt to peers and an institution, may be beneficial for ASD patients. This reflects the specific aim of AEVE: to promote the socialization and communication of ASD children to allow them to integrate into a regular school system.

**Fig. 3:**
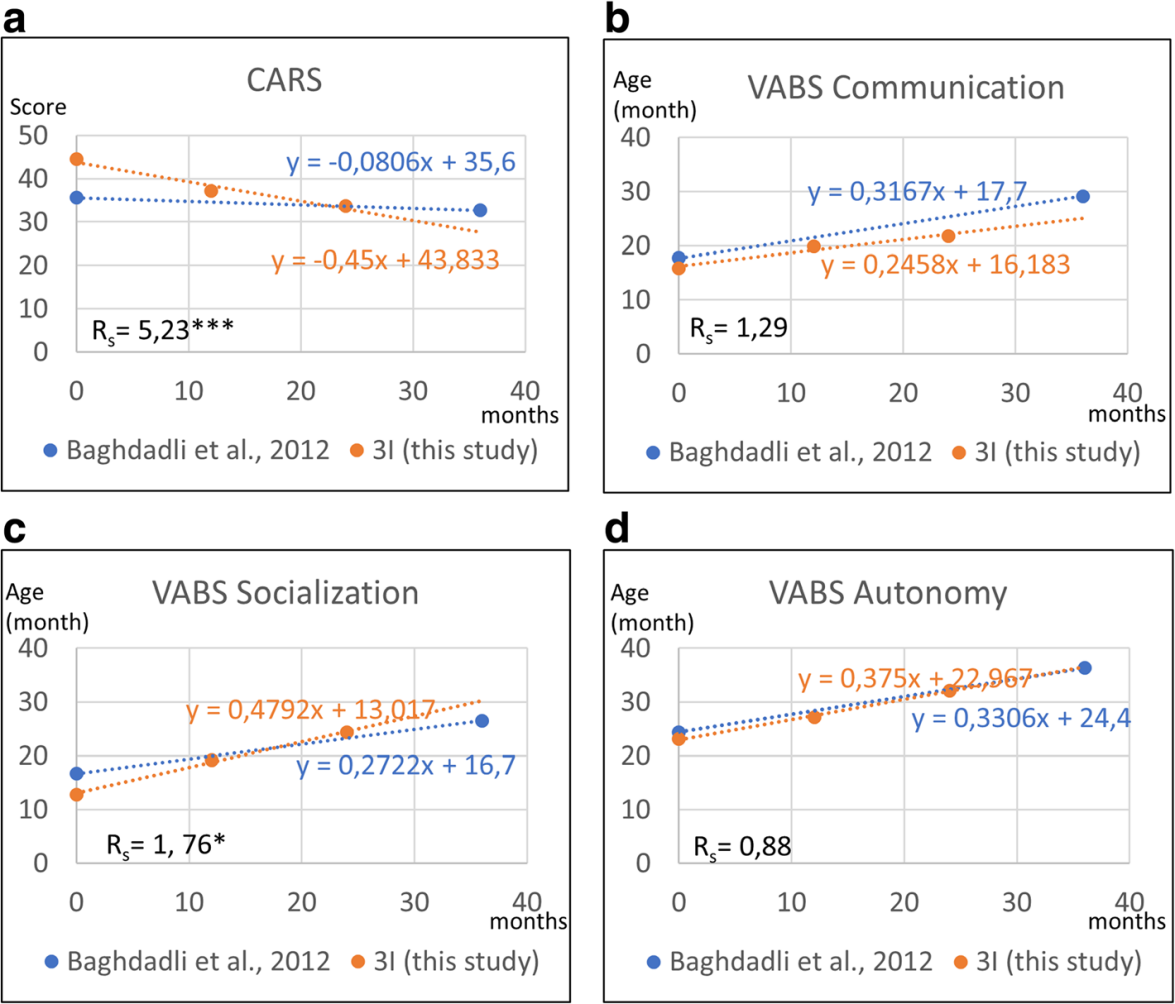
Comparison of evolution of CARS and VABS scores between 24 months of 3I methods (this study) and 36 months of standard treatment in centers according to Baghdadli et al., [48]. Evolution of the mean scores of CARS (panel **a**), VABS communication (Panel **b**), VABS socialization (Panel **c**) and VABS autonomy (Panel **d**) are represented in graphs. A linear regression was calculated for each treatment and the related equations are represented in the graphic. The ratio of the slopes (R s) indicate difference between the evolution of scores of Baghdadli et al., [48] and this study. A P-value calculated with the one sample t-test compare the distribution of the slope calculated for each subject with the mean slope of Baghdadli et al., [48]. * *p*-value < 0.05; **p-value < 0.01; *** *p*-value < 0.001

***Limitations of the study*** must, however, be noted. First, this was a prospective non-controlled study. Indeed, from an ethical and practical point of view, it was impossible to find subjects who had not received any ASD treatment for 2 years. However, in the absence of a control group, we attempted to compare our results with data from Baghdadli et al., 2012, over a 3-year period [48]. The authors used CARS and VABS tests to assess the mean developmental trajectories of autism severity and adaptive behaviours in 152 children (mean age 4.9) who were treated with various interventions (median 28 h/week, including schooling, educational therapy, physiotherapy and speech therapy) in dozens of French centres. Figure 3 shows that the slope of CARS decreases 5.23-fold more in 3i children compared to Baghdadli’s cohort (Fig. 3a). Considering changes in VABS developmental ages (in months) in 3i children compared to Baghdadli’s cohort, communication scores increased 1.29-fold less (Fig. 3b), socialization scores increased 1.76-fold more (Fig. 3C) and autonomy scores increased 1.13-fold more (Fig. 3D). These data suggest that although progress in communication and autonomy seem to be equivalent between the 3i method and various other treatments, progress in socialization may be noteworthy with the 3i method. In addition, the overall autism severity assessed by CARS seemed to decrease importantly in 3i patients compared to Baghdadli’s cohort. Another limitation of this study was the limited size of our sample. Although our previous retrospective study showed a positive evolution in 120 children (Favrot C, Saint-Georges-Chaumet Y, Saint-Georges C: Evaluation of the efficiency of the 3i method from a retrospective archive study on 120 children diagnosed with Autism Spectrum Disorders, submitted), a prospective controlled study in a larger sample is necessary. The large age range could also limit the statistical power and interpretation. However, excluding older patients would have decreased the inclusivity. Regardless, age did not seem to affect the outcomes in our study (Additional file 2: Tables S1 and S3). A larger study would be necessary to confidently state the effect of age on the 3i method’s efficiency. Finally, the 3i method is to be considered an experimental intervention in ASD. The method is still not manualized and standardized. However, 3i Method has been described in English [49], and the AEVE has developed a typical training program for psychologists enriched by filmed training materials. Hence, the replication of the 3i method is possible all over the world provided that national ASD experts are appropriately trained in France (to ensure easy recruitment and supervision of volunteers, setting-up of play rooms and monitoring of patients) and then become 3i trainers in their country. The 3i method has been already replicated in Poland at the request of the Malta Center for handicapped children in Cracow [35, 37]. One Polish ASD professional was trained in France and then provided 3i training to all professionals of the Center, where the 3i method became the primary therapy for autism. In subsequent years, the Malta Center expanded the “METODA 3i” to various locations in Poland (centers, schools, foundations in Cracow, Lublin; Warsaw, Hrubierszów and Bydgoszcz) under the conceptual and educational supervision via “AEVE Poland”.This supervision is operated through organized exchange of information and the annual visit of a French 3i expert. Moreover, the method is well known by local Universities of which many students are volunteer in 3i teams. Since September 2017 the 3i method is also replicated in Volos (Greece) by a Greek psychologist trained in France. Lastly, two teachers of a school for mentally handicapped persons in Hangzhou (China) were 3i trained in November 2017, with a view to implementing the method in this school. Thus, 3i method could be developed in various countries and represent cost-effective alternatives. Manualizing the method in the future is highly considered.

## Conclusions

This is the first prospective study to suggest the positive effects of the 3i method on behavioural and developmental skills and on ASD severity. The results presented in this study are in line with previous retrospective studies on the 3i intervention. They are also consistent with other methods using play therapy but may represent an advantage of the 3i method because it is less expensive. Further studies are necessary to support these initial results.

## Additional files

Additional file 1: Protocol accepted by the French agency for health and drug (ANSM) under the number ID-RCB 2014-A00542–45, reference: B148558–31 (DOCX 57 kb)

Additional file 2: **Table S1.** Individual results of the ADI-R, CARS, VABS, PEP-R and IMITATION (Nadel) tests for all participant of the study. **Table S2.** Mean ratio of the results between T2 an T0 of the different scores of this study of the 17 children followed at home and the 3 subjects followed in center. **Table S3.** Mean ratio of the results between T2 an T0 of the different scores of this study of the 16 children under 6 years old and the 4 subjects aged more than 7 years old. (DOCX 45 kb)

## Abbreviations

ABA: Applied behaviour analysis
AD: Autistic disorder
ADI-R: Autism diagnostic interview - revised
AEVE: *Association autisme espoir vers l’Ecole*
ASD: Autism spectrum disorders
CARS: Child autism rating scale
DIR: Individual differences and relationship-based
DQ: Developmental quotient
DSM: Diagnostic and statistical manual of mental disorders
EDT: Exchange and development therapy
ESDM: Early start denver model
ID: Intellectual disability
NIS: Nadel imitation scale
PDD-NOS: Pervasive developmental disorder-not otherwise specified
PEP-R: Psycho-educational profile – revised
TEACCH: Therapy of education of autistic and related communication handicapped cHildren
VABS: Vineland adaptive behaviour scale
