# Supplementary material for: Clinical outcomes of interactive, intensive and individual (3i) play therapy for children with ASD: a two-year follow-up study
Source: BMC Pediatr. 2018 May 12;18:165. doi: 10.1186/s12887-018-1126-7 (PMC5948870; doi:10.1186/s12887-018-1126-7)
Supplement: Supplementary file 1 — Protocol accepted by the French agency for health and drug (ANSM) under the number ID-RCB 2014-A00542–45, reference: B148558–31 (DOCX 57 kb) [file 12887_2018_1126_MOESM1_ESM.docx]

|  | **General information** |
| --- | --- |
| **1.1** | **Full title:** Evaluation of the effects of the management of children with autism spectrum disorders by the 3I method: longitudinal, retrospective and prospective follow-up over a total of two years per child**.**  **Short title:** effects of patient’s support by the 3I method |
| **1.2** | **Sponsor**: Association Autisme Espoir Vers l’Ecole (AEVE) SIREN 488 766 650 35 rue Emile Landrin 92100 Boulogne Billancourt |
| **1.3** | **Signatory on behalf of the Promoter:** Bruno de La Presle, secretary of the board of directors, 35 rue Emile Landrin 92100. bdelapresle@hotmail.fr, tel 06 14 94 06 59 |
| **1.4** | **Principal investigator**: Docteur Catherine Saint-Georges MD. PhD., child psychiatrist, 10 rue Euryale Dehaynin, 75019 Paris  **Other investigator** : Madame Bora Han, PhD. In Psychology, 9 bis rue des Bergers 75015 Paris  Place of the research: Centre Lud’éveil 3I, 57 rue Victor Hugo 92400 Courbevoie |
| **2** | **Scientific justification and general description of the research**  The 3I method (individual, intensive and interactive) is a method of developmental management of autism and pervasive developmental disorders, originally inspired by the program Son-Rise ®, developed by AEVE when The HAS (Haute Autorité de Santé: Health Hight Authority) in March 2012 ranked this method as a non-recommended method in the absence of expert consensus and scientific assessment. The HAS however expressed the wish, that innovative methods, namely the 3I method, be the subject of clinical evaluations.  The "medical device" to be evaluated consists of support of the child (or teenager) in a playroom taking into account the sensory particularities of the child to facilitate the possibility of an exchange. For the most part the support is done by nonprofessional volunteers. Volunteers are trained in the 3I method, both before the start of the care, and during monthly experience-sharing meetings, in the presence of parents, and a professional psychologist. – fully trained to the 3I method and approved by AEVE.  The play-therapy sessions, lasting one and a half hours, two in the morning and at least two in the afternoon, putting the child in contact with about thirty volunteers during the week. The sessions are child-friendly and non-directive, but the volunteers are aware of ongoing developments that they need to make. As for the therapy sessions of exchange and development (developed by Professor Barthélémy at the University Hospital of Tours), these are individual play sessions with the child in which the key words for the attitude of stakeholders are: "serenity, availability, reciprocity".  The support lasts two to three years and includes steps (or phases) described in the attached INPI Filing Brochure (AEVE1 DEC1 PJ1) |
| **2.1.a** | **The intended purpose of the medical device or in vitro diagnostic medical device, if applicable specified in the instruction leaflet or instructions for use, and that provided for research:** not applicable |
| **2.1.b** | **Contraindications, possible risks:**  The implementation of the 3I method for eight years (applied to date by more than 300 children or adolescents) has not shown any contraindications or possible risks. The intensity does not relate to the course of each session, but to the fact that the level of interaction with the child remains the same throughout the day and that the schedule of the child does not involve a timeout. During Phase 1, the child spends the most time in the "playroom", one-and-a-half hour sessions will be held with a volunteer (preferably on the same day and at the same time each day of the week), two in the morning and two or three in the afternoon. During phases 2 and 3, the child's schedule will be more diversified and oriented more towards the discovery of the outside world, prior to collective schooling. |

| 2.1.c | **Preliminary safety and performance checks:**  The security of the playroom is the responsibility of the parents; the typical equipment of the playroom recommended by the 3I method does not present any risk of physical safety (see file AEVE1 DEC1 PJ2)  The risk of maltreatment, even acts of pedophilia, can not be completely excluded, but it is very limited by the permanent presence near the playroom of a person from the family circle, which welcomes the successive volunteers and can hear what's happening in the playroom; in addition, the sessions are filmed, and any interruption of a shooting would alert the parents. |
| --- | --- |
| 2.1.d | **Summary of Training Needed by volunteers:**  The nonprofessional volunteer must be be comfortable with children and not to feel like wasting time playing with a child. Training is provided in several stages; First presentation of the method is made to people who express their interest during a research campaign of "volunteers". Second, a training is provided to new participants to present the child and its characteristics and to give first advice on how to behave in a playroom. Continuous training is then provided each month (after 4 sessions for each participant in average, based on a weekly commitment) by a psychologist, who personally ensures a weekly play session. The sessions being filmed, the permanent formation can be based on concrete examples. |
| 2.2 | **Summary of available and relevant trials:**   Empirical results obtained:  The first experiment concerned a 2-year-old child, severely affected, who was able, after three years of care, to gradually integrate normal schooling. Today, this twelve-year-old boy is in the top of his grade five class at a general education college.  Since then, a retrospective study (article in the course of writing) concerning 120 children, most of whom were between 2 and 12 years old at the beginning of care and have benefited from the 3I method continuously for 2 years shows that most of these children manage to make significant progress in the field of socialization (less isolation and better social contact) and communication (better understanding and better expression). In view of these results, empirically measured without external control, AEVE wanted to develop a research protocol to evaluate scientifically the results of the 3I method (objective of this research).  Pre-study:  A pre-study was conducted during 2013 to test the experimental setup designed for this research. Overall, the trials conducted with 14 subjects highlighted the proper functioning of the protocol (time management for home visits by the evaluator and for the administration of on-site tests, management of recording equipment during the assessments and behavioral problems that may occur in the presence of this material) and its relevance to the envisaged age of inclusion. |
| 2.3 | **Summary of Benefits and Risks:**  The expected benefits are a continuation of child or adolescent’s development through increased self-awareness that is a prerequisite for shared attention, imitation and Communication. The abilities acquired as part of the playroom are quite naturally transposable by the child in other settings.  The reservations or criticisms most frequently invoked relate to:  - a) The unprofessional nature of the volunteers,  - b) The intensive nature of the care,  - c) Deprivation of the possibility of socialization in schools  - d) The "universal" character of the 3I method |
|  | **The innovation provided by AEVE are:**  -a) The nonprofessional volunteers become experts of his child and their energy and enthusiasm from week to week are a precious asset to make the child "come into our world", under the control of the professional psychologist; the evaluation grids (file AEVE1 DEC1 PJ3) make it possible to gather observations of each participant and to obtain a faithful and precise image of the evolution of the child and, consequently, progress.  - b) No imposition of rhythm exceeding the child's endurance. If the child needs to rest, he is offered a quiet game or is allowed to sleep. When crises appear (dissatisfaction, incomprehension, frustration) the volunteer remains calm, attempts to verbalize the cause of the crisis - if he guessed it - and settles an activity to which the child ends up interested  - c) AEVE considers it is possible to bring the child to the end of the method of the 3I to a level of autonomy and capacity of learning, allowing a real inclusion in the school. This is most often prepared by home school in phase 3. And such access to school is much more beneficial for the child (and for his class).  - d) The 3I method is obviously not a panacea or a miracle method, but it can be adapted to many cases because of the flexibility of its implementation. |
| 2.4 | **Description and justification of the methods of use of the device and the duration of treatment:**  The playroom layout is designed to reduce the child's sensory perception: the light is diffused, the floor is soft, the sounds are damped, the games are stored on high shelves and the floor is is not cluttered with objects. A mirror on the wall, a swing are part of the basic equipment.  The volunteers follow each other in increments of one hour and a half (two in the morning, two or three in the afternoon). In phases 2 and 3, the child progressively left the playroom for external contacts, then for pre-school learning.  Additional care, is useful (speech therapy, psychomotricity or others) as they do not present learning modes contrary to the developmental approach 3I. Thus, assisted communication tools such as PECS are, in most cases, not recommended in the context of additional support. Likewise, learnings based on reinforcers other than mere verbal encouragement are not recommended.  The duration of 3I method usually lasts from two to three years. If the child starts from a relatively favorable level of development, it is possible (on average almost two out of ten according to the retrospective study of 120 children) that the child is admitted to class before the end of two years of care. |
| 2.5 | The research will be conducted according to the protocol with data record prior the beginning of the trial will be used for the purposes of the longitudinal study as soon as the parents have given explicit informed consent. |
| 2.6 | **Description of the population to study:**  An experimental group of 20 children with a diagnosis of Autism Spectrum Disorder who are not in school and benefiting from the 3I method between 30 and 35 hours per week will be recruited (Method 3I 'pure', without further intervention). The age of onset of care will range from approximately 2 to 12 years. Older children may be studied as well and may be the subject of separate studies. |
| 2.7 | The most recent scientific publications justifying the interest of the present research on a developmental method of management of autism are to our knowledge:  - R. Blanc et al.: Exchange Therapy and Development: A Functional Rehabilitation of Social Communication (Neuropsychiatry of Childhood and Adolescence 2013)  - Kat Houghton and others: Promoting child-initiated social-communication in children with autism: Son-Rise Intervention Effects Program (Journal of Communication Disorders 2013)  - A study of the Floortime has shown positive effects of play with the child practiced extensively by parents (A one-year prospective follow-up study of DIR / Floortime parent training intervention for pre-school children with autistic Spectrum disorders. K1, Nopmaneejumruslers K. Med Assoc Thai, 2012 Sep; 95 (9): 1184-93.  - Dawson's Early Start Denver Model study has shown clinical efficacy and even improved brain function by combining aspects of developmental behavioral therapy and spontaneous exchange aspects based on pleasure and respecting the patient's initiative. child (Dawson, G., Jones, EJH, Merkle, K., Venema, K., Lowy, R., Faja, S., ... Webb, SJ (2012) Early Behavioral Intervention Is Associated With Normalized Brain Activity in Young Children With Autism.Journal of the American Academy of Child & Adolescent Psychiatry, 51 (11), 1150-1159, doi: http://dx.doi.org/10.1016/j.jaac.2012.08.018 |
| **3** | **Goal of the research** |
|  | **Description of the main objective of the research and, if appropriate, secondary objectives:**  The main objective of this research is to evaluate the effectiveness of the 3I method by measuring the evolution of children diagnosed with autism, using recognized scales. We hypothesize that the 3I method would facilitate the development of the socio-emotional capacities of children with autism and promote the reduction of their functional and behavioral disorders.  The secondary objective of this research is to refine the evaluation of each child by studying the different domains that define autistic disorders: qualitative alterations of social interactions, qualitative alteration of communication, restricted, repetitive and stereotypical character of behavior, interests and activities.   This type of study has interests for the child because it highlights the areas in which subject makes progress, stagnates or regresses. It also makes it possible to objectify the gains and losses of development of the child with its regularities, its variability observed over time (Adrien et al., 2007). |
| **4** | **Design of the study** |
| 4.1 | **Specific statement of the main evaluation criteria and, if applicable, secondary evaluation criteria:**  4.1.1. The main evaluation criteria are:  • The development of socio-communicative skills evaluated by the imitation scale of Nadel as well as the communication and socialization scores at Vineland.  4.1.2. The secondary evaluation criteria are:  • Imitation scores and verbal cognition at PEP-R  • The severity of autistic disorders (CARS) |
| 4.2 | **Description of the research methodology, accompanied by its schematic presentation:**  Evaluating a method involves making objective comparisons before, during and after its application. To measure the impact of the 3I method on the development of the child with autism spectrum disorders, we will compare the status of the group and each subject in the group before and after the intervention. Several evaluations will be conducted over 2 years as needed (reported in terms of significant change by volunteers) but not more than 4 to avoid the effects of repeat testing.  As shown in Figure 1, the study will last during a two-year period per included child from the pre-inclusion period.   \|  \| Pre-  inclusion \| Assessment T0  (+1month) \| Assessment T1  (+12 months) \| Assessment T2  (+24 months) \| \| --- \| --- \| --- \| --- \| --- \| \| Informed consent \| x \|  \|  \|  \| \| Inclusion criteria \| x \|  \|  \|  \| \| ADI-R \| x \|  \|  \| x \| \| CARS \| x \| x \| x \| x \| \| VABS \|  \| x \| x \| x \| \| PEP-R \|  \| x \| x \| x \| \| IMITATION \|  \| x \| x \| x \|   **Pre-inclusion period:** The recruitment of the subjects will be done on the basis of the criteria of inclusion previously established. AEVE will be responsible for obtaining the diagnostic assessments (ADI-R and CARS) of the children who will be included in the study and will obtain informed consents from the responsible parents of the child. Each parent will be informed of the study by an information leaflet (see information leaflet) and may ask all questions to the site investigator and scientific managers on the site. AEVE will communicate the list of subjects to the evaluation 1 month before the beginning of the trial. The psychologist will get in touch with the subject's parents for a pre-inclusion interview (checking the inclusion criteria and making an appointment for the first assessment). The first assessment will take place one month after the first day of care. This methodological choice was made to avoid the effect of novelty on evaluation sessions. This period will allow the child to become familiar with the change (individual and intensive care that can generate situations that are not favorable to the evaluations.  **Inclusion period:** The assessments will be carried out in the place of care (either at home or in Lud'éveil) individually in the usual playroom of the child or in a suitable environment for parental maintenance.  The research methodology for this inclusion period is divided into two phases.  Phase 1 concerns the test administration phase. The evaluation sessions will be recorded via cameras installed in the game room or via an audio recorder for parental interviews.  Phase 2 : archiving of records and data collection. This is an intermediate data processing that will consist of rating and transcribing the results obtained in the different tests. A video editing work (grouping different shots into a single sequence) will be needed to facilitate the coding of social-emotional behavior during the imitation sessions. A work that will be done from the Dartfish software. The expected date of commencement of inclusions is January 1, 2013 and the expected date of completion of inclusions is April 30, 2014.  After 12 months of care, a second assessment will take place under the same conditions following the same working methodology. Intermediate data processing can be done on the data collected at 1 month of management compared to those obtained at 12 months.  Concretely and given the administrative delays related to the qualification of this research (October 2013 to February 2014) and constitution and review of the data, the study will be a longitudinal study covering two years of care per child but including a retrospective part, for data relating to a period prior to the authorization of the research and a prospective part for data subsequent to this authorization.  **End of the tests:** A last assessment identical to the previous ones will take place after 24 months of care. This period corresponds to the date scheduled for the end of the tests which will be around April 30, 2016. The end of the study will be 3 months after the end of the tests, in order to have all the necessary time to complete all the tests, analyzes and write a scientific article.  **Duration of the study of a treatment:** The result of a method is expected over 2 years, as noted by the team of Tours (Barthélémy, Hameury & Lelord, 1995) or Sally Rogers, co-author Early Start Denver (Rogers & Dawson, 2012). Given the progressive recruitment of children under the 3I.  **Evaluation sites:** at the Center de Courbevoie or at home  A 3I Awakening Center in Courbevoie hosts since January 2013, 5 children with volunteers. The places of evaluation would be this center for the 5 children or at their home, for the others. The assessments will take place in the child's usual playroom or in a place suitable for parenting interviews. In order to optimize the evaluation sessions, the playroom will be designed to exclude any element likely to deconcentrate the child during the assessment (slide, swing, mirror, ...). It will be equipped with two chairs and a table, two tripod cameras and equipment necessary for the evaluation. |
| 4.3 | **Description of assessment parameters and methods to measure, collect, analyze:**  **Rating settings:**  • The severity of autistic disorders  • The homogeneity of development  • The development of socio-communicative skills  • Adaptive behavior  Method: The evaluation of the efficiency of the 3I method will be done through the acquisition of behavioral data from the administration of the recognized test batteries. Measuring instruments sensitive enough to assess irregular development and atypical behaviors specific to ASD will be used: ADI-R (Autism Diagnostic Interview Revised), CARS (Childhood Autism Rating Scale), PEP-R (Psycho-Educational Profile Revised), VABS (Vineland Adaptive Behavior Scales) and Scale of Imitation (Jacqueline Nadel, 2012). These instruments are relatively flexible in terms of administration and make it possible to highlight the strengths and weaknesses of the individual in each of the areas, following a developmental progression of acquisitions.  Through these measurement instruments that will be administered to the child (PEP-R and Imitation scale) and from the parents (ADI-R, CARS, VABS) before, during and after the method, we will perform an analysis of performance progression during support. A global and specific statistical analysis of each GLMM (general linear mixed model) will be conducted using a quantitative method based on the scores obtained for each test, seeking a significant improvement in the scores observed. |
| 4.4 | **Description of measures taken to reduce and avoid analysis biases:**  Any validation raises methodological bias that are difficult to address. One of the questions investigators can act on is the inter-judge subjectivity for coding. Indeed, faced with varied profiles children with atypical behavior (which are sometimes very difficult to interpret), the quotes can vary from one judge to another. To avoid this analysis bias, an objective analysis grid will serve as a guide and a double coding will be done on the evaluation records. Thus we will proceed to a calculation of kappa coefficient to see the degree of agreement between several judges.  A key question that arises is: what to attribute a significant difference to? In two years, young children with autism change a lot, as do all children: longitudinal follow-ups indicate that children with the same initial profile diverge, with some of them making great progress while others stagnate or even regress (Pry & Darrou , 2009). To resolve this proble, since a control group could not be constituted, the evolution will be compared to the data available in the literature, notably (if possible) ongoing study also using PEP, CARS, Vineland and ADI-R (search workshop-class Preaut). It will take a massive superiority of the results of the method of 3I to be attributed as such the success eventually achieved. |
| **4.5** | **Detailed description of how to use the device:**  The implementation of the device assumes:  - The equipment of the 3I game room (file AEVE1 DEC1 PJ2)  - Research and selection of non-professional workers, mentioned in 2.1.d (there is usually a great diversity of stakeholders, each of whom brings to the child a way to play and their own aptitudes and interests most of them are women)  - The choice of a psychologist having the approval for a 3I care,  - The organization of the weekly schedule of the volunteers,  - The game in turn session in individual with the child,  - The note taking by the volunteer on the course of his session, with the possible mention of passages to be reviewed by the psychologist,  - The weekly session of the child with the psychologist,  - Holding of the monthly meeting of experience sharing, evaluation according to the developmental evaluation grid 3I skills and know-how, (file AEVE1 DEC1 PJ3) allowing a sharing of feeling and experience and to identify progress to be encouraged. |
| **4.6** | **Expected duration of participation, chronology description:**  The total duration of participation of a child or teenager benefiting from the 3I method will be approximately 3 hours for each evaluation (1h for the Imitation session, 15 min break and 1h45h for the PEP). The total duration of the parent's participation will be approximately 3 hours for each evaluation (30min of pre-inclusion telephone interview, 1h for CARS and 1h30 for VABS). To this is added 4h maintenance for the ADI-R in pre-inclusion period (2h) and end of tests (2h). Over a period of 2 years of follow-up, the total duration of participation (of the child and the parent) in the specific assessments will be around 22 hours.  The duration of care as such (already performed routinely on many children) will be 30 to 35 hours per week over 2 years. |
| **4.7** | **Description of the definitive or temporary cessation rules:**  The dissolution or cessation of activity of the association Autisme Espoir Vers l'Ecole would constitute a case of permanent termination of this research as well as a ban on the method of 3I by the competent authorities.  The worsening observed at intermediate assessments in at least 1/3 of children, or the occurrence of serious adverse effects motivating discontinuation of treatment in ¼ of children, would motivate a decision to discontinue the study. |
| **4.8** | Measures implemented to maintain the blind and procedures for lifting the blind, the case  Due: Not applicable |
| **4.9** | **Identification of all "source" data to be collected directly in the observation books:**  The source data are: medical file, demographic data, family environment (couple or single parent, siblings, socio-economic level, academic level, health, family climate), audio and video recording of evaluations, rating, transcription and synthesis of results. |
| **5** | **Selection and exclusion of people from research** |
| **5.1** | **Inclusion criteria**  **•** Child and adolescent diagnosed with ASD (criteria: ADI-R and CARS)  • Parental agreement and signed informed consent  • Child who starts the 3I method from January 2013  • Age between 2 years and 12 years at the beginning of the treatment 3I for the experimental group  • Consistent application of the method (adequate playroom, number of volunteers and number of hours of stimulation between 30-35h per week)  • Out-of-school child and adolescent who benefits from the 3I 'pure' method, without any other major intervention.  • Child with French in mother tongue |
| **5.2** | **Criteria of non-inclusion**  • Associated pathology (epilepsy, Rett syndrome ...)  • Child with Asperger syndrome  • Family situation that does not make it possible to consider taking care of 3I in the long term  • Children who do not do 100% of 3I method (part-time support)  • Child receiving other type ABA care, TEACCH |
| **5.3** | **Premature stop procedure:**  The regulation concerning the events and undesirable effects will be respected.  In the event of an undesirable effect (lasting aggravation of behavioral disorders)  In the case of significant improvement indicating partial or total re-enrollment  In case of unilateral decision of the family to leave the research and / or care 3I  In the first two cases, the investigator and the collaborating investigator, possibly solicited by a referent psychologist of the child, will decide on the premature exit of the study, having, as far as possible, carried out the tests so to limit the missing data (and also in the third case). |
| **6** | **Treatment other than the device:**  Additional interventions in speech therapy, psychomotricity, occupational therapy and other interventions that may require technical actions that are not the responsibility of 3I volunteers may be associated. These interventions will be recorded to analyze if they can biased the interpretation of the results. |
| **7** | **Performance evaluation:** |
| **7.1** | **Description of performance evaluation parameters:**  • The severity of autistic disorders (CARS)  • Homogeneity of development (PEP)  • The development of socio-communicative skills (IMITATION)  • Adaptive behavior (VABS) |
| **7.2** | **Methods and schedule for measuring, collecting and analyzing evaluation parameters:**  The quantitative methods used to measure performance changes will consist in comparing the results obtained at the different assessments at different periods of care (T + 1 month, T + 12 months, T + 24 months). The collection and analysis of intermediate data will be carried out progressively as the evaluations are carried out. The first analysis will be conducted at the end of the second evaluation period (T + 12 months), we will compare the data from all these tests, at T + 1months. period T + 12 months. A second analysis will be conducted at the end of the tests to compare the performance of the method between the three measurement periods (T + 1 month vs. T + 12 months vs. T + 24 months).  Through PEP-R (Schopler, 2005), we will conduct a performance analysis based on an assessment and direct observation of the child. The analysis will focus on two types of data collected: 1) the age of global and specific development corresponding to different areas of development (Imitation, Perception, fine motor skills, gross motricity, hand-hand coordination, cognitive performance, verbal cognition) and And 2) behavioral observations (relationships and affect, play and interest in the material, sensory responses, and language) collected during the assessment sessions. This tool, which gives a global and specific level of development for each evaluated domain, makes it possible to see the homogeneity of the development. The more the development becomes homogeneous during the 2 years of care, the better the performance of the 3I method.  Via the Scale of Imitation (Nadel, 2012), we will perform a detailed analysis of socio-communicative skills. This tool makes it possible to evaluate the level of the preverbal communication, in particular that of the imitation. It consists of three scales: spontaneous imitation, recognition to be imitated and imitation on request. The analysis of the scores obtained in these three sub-scales will make it possible to better follow the effect of this method on the development of nonverbal communication in very young or non-verbal subjects. An analysis of socio-emotional behavior (CPSE) based on a behavioral coding grid will measure the impact of the 3I method on the evolution of social-emotional behaviors (positive vs. negative). The higher the imitation scores and the number of positive social-emotional behaviors increase over the 2 years of care, the better the 3I method's performance.  These tests will be accompanied by 3 semi-structured interviews with parents (ADI-R, CARS, VABS). These are multidimensional assessments of learning and dysfunctions in everyday life.  The ADI-R which is a diagnostic assessment tool focuses on the description and history of child development in the three domains: quality of social interactions, communication and language, narrow interests and stereotyped behaviors. A change in scores on this parent questionnaire might reflect the impact of the method.  CARS, which is also a diagnostic tool, will allow us to assess the intensity of autistic disorders and their evolution during the trial. The 15 domains usually disturbed in autism are evaluated there: social relations, imitation, emotional response, use of the body, use of objects, adaptation to change, visual responses, auditory responses, taste-smell-touch (responses and modes of exploration), fear and anxiety, verbal communication, nonverbal communication, level of activity, intellectual level and homogeneity of intellectual functioning and general impression. The lower the overall score during trail, the better the performance of the 3I method.  The VABS which is a social adaptation assessment tool makes it possible to analyze, through the scores converted into developmental age (referred to mean equivalent ages of the normal sample), the impact of the 3I method on the development of socialization, communication, and the autonomy of everyday life. The more the difference between the chronological age of the subject and his age of development decreases during the management, the better the performance of method 3I. |
| **8** | **Security assessment**  The 3I method is carried out in a family setting, with the exception of the center of Lud'éveil Courbevoie for five children. Child safety is ensured mainly by his parents. The equipment in the playroom provides a camera through which all sessions can be viewed.  The risk of exposure to pedophilia by volunteers is not to be neglected; it is lessened by the presence of another person in the immediate vicinity of the playroom, and by the recording of the camera, which any interruption would raise the mistrust of the parents.  The occurrence of any events or adverse effects, within the meaning of § 5.3 will be notified by the 3I psychologist assigned to the child to the investigator. |
| **9** | **Statistisques :** |
| **9.1** | **Description of planned statistical methods and timing of interim analyzes:**  Statistical methods: It will compare the mean of the scores or the ages of developments of the cohort obtained with the different tests of the evaluation carried out at T0, T1, T2. Once the conditions of application of the parametric tests are verified we will apply a general linear mixed model (GLMM) to obtain a better statistical power while taking into account the individual variations of the subjects (subject effect). Additional analyzes will take into account the age group of the child in covariate, as well as the severity of the disorders, and the level of language (evaluated at Vineland). Individual studies may be carried out.  Independent variable:  • Time: evaluation at T0, T1, T2  Dependent variables for each test:  • CARS: total score  • VABS: age of development by domain (4)  • PEP-R: overall development age, age of development by domain (7) IMITATION: score by domain (3), number of socio-emotional behavior (positive vs negative)  In addition, we will analyze the profiles of each subject to evaluate areas of evolution (case study).  Calendar of interim analyzes:  • Intermediate analysis on T1 tests compared to T0  • Final analysis on T2 tests compared to T0 |
| **9.2** | **Expected number of subjects to include and statistical justification:**  About 20 subjects in the experimental group 2 years-12 years to perform parametric tests. |
| **9.3** | **Degree of signification:**  The main effects and interactions will be considered significant if the p-value is lower than the risk α = 0.05 |
| **9.4** | **Statistical criteria for stopping the search:**  If the interim analysis at T1 showed a significant deterioration of the measures of functioning of the children |
| **9.5** | **Method of taking into account missing data:**  • To take into account the missing data we will use alternative sources of information. For example, in the case where the child does not realize an item (for lack of interest) this information will be collected from the child's parents or psychologists. This information should be verified on written accounts of volunteers or video recordings of play sessions. If there is missing data in one of the tests, the subject data concerning the test in question will be excluded from the analysis.  • In the case of premature termination of the method (unilateral decision of the family) tests will be made to estimate the evolution of the child, even outside the scheduled timing for assessments. If it is impossible to carry out these tests, we will be able to use the last films of the working sessions to rate CARS and Vineland. In all cases, those lost to follow-up will have to be taken into account in the interpretation of the results because they could constitute a bias (children progressing insufficiently being more likely to stop care prematurely). |
| **9.6** | **Change management at the initial statistical level**  A priori not applicable |
| **10** | **Right of access to data and source documents**  The right of access to the data and source documents will be strictly reserved to the investigator, to the collaborator of the investigator and to the person in charge of the quality control (cf §11) |
| **11** | **Quality control and quality assurance**  The investigator's collaborator will work under the control of the investigator. The relatively repetitive nature of the tests performed did not seem to require the intervention of a third party in quality control and quality assurance. |
| **12** | **Ethical considerations**  Respect for the privacy of the families whose children will participate in the study is a key concern of the Promoter of this research.  The application for research authorization will be made to the CNIL under the responsibility of the Promoter.  To date, no data available suggests that children or adolescents participating in this research are associated with 3I management.  The realization of this research is an essential condition for improving the administrative and financial management of families choosing support 3I |
| **13** | **Processing and preservation of documents and research data**  All the files of the study will be archived for a period of 15 years, under the responsibility of the Promoter using, preferably and subject to the agreement of its director, the means that the laboratory of use of digital techniques de la Villette could put at his disposal.   The promoter of the study will subcontract, in the absence of agreement with the laboratory above, to an outside company, such as Archiveco, for a period of 15 years, the storage of the following documents:. - Protocol with annexes, amendments; - Consent Forms signed and initialed. - Information forms on experimentation. - Observation books (originals) with additional documents. - Follow-up document of the clinical study. - All administrative documents and correspondence related to the study. - Data acquired (test results) - Study report. |
| 14 | **Financing and insurance**  Funding for the pre-study was provided in 2013 by a grant from the Bettencourt Schueller Foundation.  AEVE had 45,000 euros in cash on 1 January 2014 (30,000 euros from the Bettencourt Schueller Foundation and 15,000 euros from the Deehcq Institut de France Foundation). The Bettencourt Schueller Foundation is also committed to pay a grant balance of € 10,000 for this project. The cost of the 2014 2016 project is estimated at € 70,000 and AEVE has sufficient capital to cover the unfinanced balance if no new grant is obtained.  AEVE will present a certificate of insurance covering his civil liability as a promoter as well as for the investigator, intervening free of charge, and the collaborator of the investigator, acting as a self-entrepreneur. |
| **15** | **Publication rules:**  All data collected during this study is the property of the sponsor of the study and can not be communicated to any third party without the written consent of the principal investigator or associate and the sponsor.  Any publication or communication (oral or written) will be decided by mutual agreement between the investigators and will respect the international recommendations: "Uniforms Requirements for Manuscripts Submitted to Biomedical Journals"  (Http://www.cma.ca/publications/mwc/uniform.htm)  In all publications related to the study, the sponsor will appear in the acknowledgments. |
| **16** | **List of annexes :**  - AEVE1 DEC1 PJ1 Description of the 3I method filed with the INPI,  - AEVE1 DEC1 PJ2 Descriptive note of the game room 3I  - AEVE1 DEC1 PJ3 Evaluation grid 3I |

Signed on April 23, 2014

The secretary of the board of directors of AEVE

Bruno de La Presle
