# Supplementary material for: Clinical outcomes of interactive, intensive and individual (3i) play therapy for children with ASD: a two-year follow-up study
Source: BMC Pediatr. 2018 May 12;18:165. doi: 10.1186/s12887-018-1126-7 (PMC5948870; doi:10.1186/s12887-018-1126-7)
Supplement: Supplementary file 2 — Table S1. Individual results of the ADI-R, CARS, VABS, PEP-R and IMITATION (Nadel) tests for all participant of the study. Table S2. Mean ratio of the results between T2 an T0 of the different scores of this study of the 17 children followed at home and the 3 subjects followed in center. Table S3. Mean ratio of the results between T2 an T0 of the different scores of this study of the 16 children under 6 years old and the 4 subjects aged more than 7 years old. (DOCX 45 kb) [file 12887_2018_1126_MOESM2_ESM.docx]

| **Patient** | Age | **PEP-R**  **Imitation** | | | **PEP-R**  **Perception** | | | **PEP-R Fine**  **motor skills** | | | **PEP-R Global**  **motor skills** | | | **PEP-R Oculo-**  **manual** | | | **PEP-R**  **Cognitive** | | | **PEP-R T0**  **Verbal** | | | **VABS**  **Communication** | | | **VABS**  **Autonomy** | | | **VABS**  **socialization** | | | **Imitation score**  **Nadel** | | | **CARS** | | | **ADIR**  **Interaction** | | | **ADIR**  **Communication** | | | **ADIR Sterotypy** | | |
| --- | --- | --- | --- | --- | --- | --- | --- | --- | --- | --- | --- | --- | --- | --- | --- | --- | --- | --- | --- | --- | --- | --- | --- | --- | --- | --- | --- | --- | --- | --- | --- | --- | --- | --- | --- | --- | --- | --- | --- | --- | --- | --- | --- | --- | --- | --- |
|  |  | **T0** | **T1** | **T2** | **T0** | **T1** | **T2** | **T0** | **T1** | **T2** | **T0** | **T1** | **T2** | **T0** | **T1** | **T2** | **T0** | **T1** | **T2** | **T0** | **T1** | **T2** | **T0** | **T1** | **T2** | **T0** | **T1** | **T2** | **T0** | **T1** | **T2** | **T0** | **T1** | **T2** | **T0** | **T1** | **T2** | **T0** | **T1** | **T2** | **T0** | **T1** | **T2** | **T0** | **T1** | **T2** |
| P12 | 2,8 | 19 | 19 | NA | 18 | 17 | NA | 20 | 19 | NA | 19 | 24 | NA | 24 | 39 | NA | 9 | 24 | NA | 8 | 17 | NA | 9 | 15 | NA | 14 | 22 | NA | 10 | 19 | NA | 5,5 | 11,5 | NA | 53 | 34 | NA | 23 | 15 | NA | 12 | 7 | NA | 8 | 8 | NA |
| P16 | 2,8 | 16 | 17 | 17 | 25 | 38 | 38 | 18 | 30 | 28 | 22 | 27 | 32 | 19 | 28 | 32 | 14 | 16 | 14 | 17 | 17 | 9 | 11 | 16 | 14 | 22 | 24 | 23 | 12 | 18 | 18 | 8,0 | 5,0 | 9,5 | 46 | 37 | NA | 21 | 16 | 20 | 12 | 8 | 7 | 3 | 4 | 3 |
| P11 | 2,8 | 32 | 41 | 68 | 48 | 63 | 68 | 28 | 39 | 60 | 27 | 37 | 62 | 36 | 39 | 64 | 35 | 50 | 50 | 28 | 52 | 48 | 36 | 40 | 32 | 25 | 35 | 34 | 23 | 33 | 34 | 11,5 | 14,0 | 25,0 | NA | NA | NA | 24 | NA | NA | 13 | NA | NA | 6 | NA | NA |
| P09 | 2,9 | 15 | 22 | 22 | 17 | 17 | 38 | 18 | 18 | 30 | 18 | 27 | 30 | 19 | 19 | 32 | 9 | 11 | 13 | 17 | 17 | 17 | 11 | 14 | 16 | 10 | 13 | 18 | 8 | 12 | 18 | 5,5 | 10,0 | 12,5 | 54 | 41 | 35 | 24 | 17 | 12 | 14 | 13 | 10 | 8 | 4 | 3 |
| P01 | 3,3 | 19 | 24 | 15 | 21 | 31 | 25 | 30 | 30 | 25 | 46 | 46 | 19 | 36 | 36 | 20 | 15 | 15 | 11 | 17 | 17 | 8 | 14 | 15 | 16 | 21 | 23 | 24 | 12 | 17 | 14 | 7,5 | 9,0 | 8,0 | 44 | NA | NA | 18 | NA | NA | 9 | NA | NA | 3 | NA | NA |
| P15 | 3,3 | 47 | 54 | 68 | 25 | 48 | 64 | 39 | 43 | 72 | 30 | 46 | 61 | 24 | 32 | 69 | 31 | 42 | 58 | 35 | 48 | 55 | 25 | 37 | 48 | 15 | 27 | 50 | 13 | 23 | 67 | 19,0 | 32,0 | 29,5 | 37 | 33 | 25 | 20 | 15 | 10 | 11 | 12 | 3 | 3 | 7 | 3 |
| P02 | 3,4 | 30 | 24 | 21 | 18 | 18 | 30 | 25 | 29 | 28 | 30 | 32 | 32 | 20 | 24 | 16 | 12 | 14 | 13 | 17 | 17 | 8 | 5 | 12 | 13 | 14 | 19 | 22 | 5 | 12 | 10 | 6,5 | 10,0 | 14,0 | 46 | 39 | 36 | 25 | 17 | 13 | 13 | 12 | 11 | 8 | 4 | 2 |
| P18 | 4,0 | 10 | 15 | 32 | 12 | 12 | 38 | 22 | 22 | 30 | 20 | 20 | 30 | 16 | 16 | 24 | 9 | 9 | 10 | 17 | 17 | 17 | 11 | 13 | 15 | 16 | 18 | 20 | 9 | 15 | 24 | 6,5 | 7,5 | 14,5 | 42 | 38 | 31 | 16 | 8 | 5 | 13 | 10 | 8 | 8 | 5 | 4 |
| P03 | 4,2 | 21 | 15 | 19 | 25 | 31 | 37 | 34 | 34 | 38 | 30 | 27 | 32 | 16 | 24 | 27 | 14 | 9 | 12 | 19 | 17 | 6 | 6 | 9 | 12 | 19 | 23 | 24 | 3 | 6 | 9 | 5,5 | 5,0 | 7,5 | 46 | 39 | 37 | 23 | 17 | 16 | 7 | 9 | 9 | 4 | 7 | 5 |
| P04 | 4,3 | 18 | 24 | 19 | 30 | 38 | 30 | 25 | 34 | 27 | 27 | 32 | 36 | 24 | 28 | 27 | 14 | 14 | 15 | 17 | 17 | 16 | 13 | 14 | 16 | 20 | 22 | 38 | 8 | 10 | 17 | 3,0 | 9,5 | 7,5 | 51 | 33 | 29 | 21 | 9 | 4 | 9 | 6 | 6 | 10 | 2 | 3 |
| P20 | 4,5 | 21 | 33 | 29 | 38 | 64 | 48 | 34 | 43 | 47 | 37 | 61 | 45 | 40 | 64 | 36 | 24 | 27 | 25 | 17 | 17 | 18 | 6 | 11 | 14 | 23 | 27 | 37 | 10 | 18 | 30 | 8,0 | 15,0 | 10,5 | 46 | 36 | 31 | 20 | 11 | 9 | 14 | 6 | 2 | 7 | 4 | 1 |
| P05 | 4,7 | 40 | 36 | NA | 20 | 48 | NA | 29 | 38 | NA | 29 | 61 | NA | 19 | 32 | NA | 13 | 29 | NA | 22 | 24 | NA | 15 | 18 | NA | 27 | 33 | NA | 15 | 23 | NA | 10,5 | 22,0 | NA | 42 | 30 | NA | 25 | 10 | NA | 12 | 12 | NA | 5 | 7 | NA |
| P17 | 4,7 | 21 | 30 | 21 | 25 | 38 | 30 | 25 | 25 | 21 | 26 | 37 | 24 | 16 | 24 | 20 | 14 | 16 | 9 | 17 | 17 | 18 | 11 | 14 | 16 | 16 | 18 | 23 | 10 | 19 | 17 | 8,5 | 10,5 | 15,0 | 40 | 36 | NA | 30 | 13 | NA | 14 | 8 | NA | 10 | 5 | NA |
| P19 | 5,3 | 19 | 19 | 37 | 21 | 21 | 37 | 15 | 28 | 33 | 27 | 46 | 60 | 24 | 24 | 28 | 14 | 14 | 17 | 17 | 8 | 19 | 11 | 14 | 14 | 19 | 19 | 21 | 7 | 12 | 20 | 7,0 | 10,5 | 13,5 | NA | 54 | 41 | 24 | 17 | 15 | 12 | 10 | 6 | 7 | 8 | 6 |
| P06 | 5,3 | 40 | 68 | 68 | 58 | 48 | 63 | 42 | 42 | 58 | 61 | 61 | 60 | 58 | 63 | 68 | 46 | 44 | 57 | 46 | 50 | 52 | 25 | 32 | 37 | 34 | 39 | 43 | 21 | 30 | 38 | 12,5 | 21,0 | 14,5 | 42 | NA | 29 | 24 | NA | 7 | 18 | NA | 9 | 11 | NA | 7 |
| P07 | 5,7 | 36 | 46 | 68 | 38 | 64 | 63 | 72 | 72 | 58 | 60 | 61 | 61 | 48 | 63 | 47 | 37 | 44 | 49 | 30 | 40 | 43 | 29 | 37 | 38 | 32 | 39 | 56 | 24 | 31 | 38 | 13,5 | 13,5 | 21,0 | 44 | 34 | 26 | 27 | 13 | 8 | 8 | 12 | 7 | 4 | 3 | 1 |
| Mean | 4,0 | 25,3 | 30,4 | 36,0 | 27,4 | 37,3 | 43,5 | 29,8 | 34,1 | 39,6 | 31,8 | 40,3 | 41,7 | 27,4 | 34,7 | 36,4 | 19,4 | 23,6 | 25,2 | 21,3 | 24,5 | 23,9 | 14,9 | 19,4 | 21,5 | 20,4 | 25,1 | 30,9 | 11,9 | 18,6 | 25,3 | 8,7 | 12,9 | 14,5 | 44,9 | 37,0 | 31,9 | 22,8 | 13,7 | 10,8 | 11,9 | 9,6 | 7,1 | 6,6 | 5,2 | 3,5 |
| P14 | 7,3 | 24 | 27 | 32 | 14 | 14 | 38 | 11 | 22 | 30 | 22 | 22 | 27 | 12 | 12 | 28 | 13 | 13 | 12 | 17 | 17 | 8 | 11 | 14 | 15 | 18 | 19 | 23 | 12 | 24 | 18 | 7,5 | 14,0 | 12,5 | 53 | 40 | 38 | 26 | 14 | 12 | 12 | 13 | 10 | 4 | 3 | 3 |
| P10 | 8,3 | 15 | 26 | NA | 30 | 38 | NA | 26 | 22 | NA | 22 | 46 | NA | 24 | 32 | NA | 21 | 22 | NA | 17 | 17 | NA | 11 | 13 | NA | 29 | 28 | NA | 10 | 12 | NA | 3,0 | 4,5 | NA | 40 | 35 | NA | 22 | 17 | NA | 14 | 7 | NA | 3 | 3 | NA |
| Mean | 7,8 | 19,5 | 26,5 | 32,0 | 22,0 | 26,0 | 38,0 | 18,5 | 22,0 | 30,0 | 22,0 | 34,0 | 27,0 | 18,0 | 22,0 | 28,0 | 17,0 | 17,5 | 12,0 | 17,0 | 17,0 | 8,0 | 11,0 | 13,5 | 15,0 | 23,5 | 23,5 | 23,0 | 11,0 | 18,0 | 18,0 | 5,3 | 9,3 | 12,5 | 46,0 | 37,0 | 38,0 | 24,0 | 15,5 | 12,0 | 13,0 | 10,0 | 10,0 | 3,5 | 3,0 | 3,0 |
| P08 | 12,4 | 23 | 36 | 46 | 38 | 48 | 63 | 39 | 42 | 58 | 46 | 46 | 61 | 36 | 48 | 67 | 27 | 38 | 49 | 36 | 48 | 51 | 27 | 29 | 39 | 38 | 39 | 49 | 27 | 30 | 25 | 15,5 | 21,5 | 20,5 | 39 | 31 | NA | 25 | 16 | 15 | 22 | 14 | 9 | 7 | 1 | 1 |
| P13 | 14,4 | 46 | 54 | 68 | 49 | 64 | 64 | 60 | 72 | 72 | 46 | 60 | 61 | 64 | 63 | 68 | 66 | 58 | 57 | 48 | 54 | 52 | 28 | 30 | 32 | 50 | 56 | 54 | 17 | 20 | 35 | 26,5 | 22,0 | 12,5 | 42 | 38 | 33 | 23 | 11 | 11 | 20 | 12 | 12 | 9 | 7 | 5 |
| Mean | 13,4 | 34,5 | 45,0 | 57,0 | 43,5 | 56,0 | 63,5 | 49,5 | 57,0 | 65,0 | 46,0 | 53,0 | 61,0 | 50,0 | 55,5 | 67,5 | 46,5 | 48,0 | 53,0 | 42,0 | 51,0 | 51,5 | 27,5 | 29,5 | 35,5 | 44,0 | 47,5 | 51,5 | 22,0 | 25,0 | 30,0 | 21,0 | 21,8 | 16,5 | 40,0 | 34,5 | 32,5 | 24,0 | 13,5 | 13,0 | 21,0 | 13,0 | 10,5 | 8,0 | 4,0 | 3,0 |

**Supplementary file 2 - table 1**

**Supplementary file 2 - Table 2**

| **Test** | **mean ratio T2/T0**  **"home"** | **Moy ratio T2/T0**  **"center"** | **P-value** |
| --- | --- | --- | --- |
| **PEP-R Imitation** | 1,48 | 1,32 | 0,832277 |
| **PEP-R Perception** | 1,73 | 1,38 | 0,491269 |
| **PEP-R Fine motor skills** | 1,42 | 1,23 | 0,689474 |
| **PEP-R Global motor skills** | 1,46 | 1,24 | 0,750642 |
| **PEP-R Oculo-manual**  **development** | 1,43 | 1,56 | 0,396390 |
| **PEP-R Cognitive performance** | 1,29 | 1,25 | 0,842105 |
| **PEP-R T0 Verbal cognition** | 1,08 | 0,89 | 0,395498 |
| **Imitation score Nadel** | 1,66 | 1,73 | 0,921053 |
| **VABS Communication** | 1,47 | 1,56 | 0,671534 |
| **VABS Autonomy** | 1,46 | 1,48 | 0,525203 |
| **VABS socialization** | 2,08 | 2,02 | 0,873751 |
| **CARS** | 0,75 | 0,73 | 0,875129 |
| **ADIR Interaction** | 0,57 | 0,50 | 0,915637 |
| **ADIR Communication** | 0,66 | 0,79 | 0,750004 |
| **ADIR Sterotypy** | 0,72 | 0,56 | 0,554734 |

Mean ratio of the results between T2 an T0 of the different scores of this study of the 17 children followed at home and the 3 subjects followed in center.

**Supplementary file 2 - table 3**

| **Test** | **Mean ratio T2/T0 3-5**  **years** | **Mean ratio T2/T0 >7**  **years old** | **P-value** |
| --- | --- | --- | --- |
| **PEP-R Imitation** | 1,41 | 1,64 | 0,237371 |
| **PEP-R Perception** | 1,66 | 1,74 | 0,705351 |
| **PEP-R Fine motor skills** | 1,35 | 1,57 | 0,68194 |
| **PEP-R Global motor skills** | 1,41 | 1,49 | 0,740669 |
| **PEP-R Oculo-manual**  **development** | 1,40 | 1,65 | 0,368643 |
| **PEP-R Cognitive performance** | 1,31 | 1,16 | 0,494118 |
| **PEP-R T0 Verbal cognition** | 1,06 | 0,99 | 0,812633 |
| **Imitation score Nadel** | 2,23 | 1,42 | 0,048452 |
| **VABS Communication** | 1,54 | 1,28 | 0,236841 |
| **VABS Autonomy** | 1,54 | 1,15 | 0,118833 |
| **VABS socialization** | 2,23 | 1,42 | 0,072386 |
| **CARS** | 0,73 | 0,80 | 0,184727 |
| **ADIR Interaction** | 0,55 | 0,58 | 0,67045 |
| **ADIR Communication** | 0,70 | 0,59 | 0,367738 |
| **ADIR Sterotypy** | 0,72 | 0,61 | 0,666165 |

Mean ratio of the results between T2 an T0 of the different scores of this study of the 16 children under 6 years old and the 4 subjects aged more than 7 years old.
